# Spirituality and mental health: Reflections of past, applications in present, projections for future

**Published:** 2009

**Authors:** B. R. Ravi Shankar Rao

**Affiliations:** Prof. of Psychiatry, M. S. Ramaiah Medical College, Bangalore, India


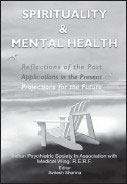


In this remarkable book 53 authors have, over 39 chapters, given us different perspectives on Spirituality and Mental Health. The canvas that is painted has a wide range, depth, color and variety.

Mental health has to be seen in its cultural context. Western psychology, psychotherapy and concepts of normality are linked to the history of Christianity and philosophically to reductionist Cartesian mind-body dualism. In contrast, Ayurveda and Yoga propound a holistic approach. In recognition of these roots the World Health Assembly, in 1999, added ‘spiritual well being’ to the WHO definition of health as secular medical science appeared mechanized and dehumanized.

The history of Indian spirituality is traced from the Vedic period through the Upanishads, Bhagvad Gita and to Ayurveda through classics like Charaka and the Sushrutha samhitas. They provide insights into concepts of personality types, causation of mental illness and its treatment. In addition to the formation of the physical, emotional and intellectual personality is the uniqueness of the Atman in creating the spiritual personality. The cognitive restructuring done by Lord Krishna on Arjuna by taking Him through the Yoga of Jnana, Bhakti and Karma provides a holistic framework conducive to freedom from psychological conflicts.

Vipassana, a very ancient meditation technique finds reference in the Rig Veda and is the quintessence of Buddha's teaching. Also known as ‘insight meditation’ it is a technique of introspection and self exploration leading to self transformation and a refinement of awareness. Preksha Dhyan, a Jaina adaptation of Vipassana, is a profound perception of the subtle innate phenomena of consciousness. In this an intuitive and discursive contemplation is followed by therapeutic thinking while in Tantra the physical union transforms into a spiritual union to attain a cosmic spiritual consciousness.

The five pillars of Islam along with the code of conduct define the basic philosophy that leads to group adherence and improved code of attitudes and behavioral norms.

An acute need for a different lexicon is felt when one discusses issues straddling different systems-Indian spiritual and modern scientific. When one tries to understand the matters of faith through reason only approximations are possible. Despite this, the spiritual dimension of holistic medicine can be integrated in current health care. Training and education of health care workers would thus require that it is an experiential process and relevant to the work they do. Spiritually Augmented Cognitive Behavior Therapy (SCABT) is a meaning based therapy. Used as an adjunct, it is an important part of caring for the whole person that is typically found wanting in the biomedical model.

The foundation of moral, ethical and spiritual development is laid in the pre school years. Through exposure to nature, creative arts etc., a spiritual consciousness develops which helps in being grounded internally-a major contributor to overall happiness.

Evidence points to a need to maintain mutual respect and for coordination between traditional healers and their methods and modern psychotherapeutic techniques. Similarly, tracing the historical links between religion and psychiatry, in a multi-faith society like the UK, a need for a coordinated and mutually respectful approach is felt. In the Indian context integrating indigenous knowledge and spirituality with biomedicine would lend a holistic dimension to mental health delivery. In Pakistan there is a paucity of trained mental health professionals and hence the role played by health providers based on spiritual and religious beliefs is highlighted.

Various regions of the brain particularly the prefrontal and anterior cingulate cortex are observed to undergo changes in different religious/spiritual practices. This has been studied using various neuro imaging techniques. The neurobiology, neurochemistry and neurophysiology of meditation have been studied and evidence of its usefulness in psychiatry and directions for the future is discussed.

It must have been a formidable task to meaningfully compile articles on a subject like spirituality, the definition of which is not clear. While appreciating that we run the risk of reductionism, incorporating spirituality into mental health care requires different perspectives. Spiritual experience is holistic and understanding spirituality is being spiritual. ‘The world of spirit begins where logic ends. Logic is limited by the boundaries of reason which is further limited by the individual's intellect. Most speak and comprehend only the tongue of logic’ (Yogi Ashwini).

It is obvious this book is the result of Avdesh Sharma's *sadhana*.

